# 

**Published:** 1904-01

**Authors:** 


### LIST OF CONTRIBUTORS TO VOLUME XXV.

Ainsworth, George C., D.D.S.
Allen, Freeman, M.D.
Andrews, R. R., A.M., D.D.S.
Anema, R., D.D.S.
Arrington, B. F., D.D.S.
Baker, H. A., D.D.S.
Baker, Lawrence W., D.M.D.
Baldwin, A. E., M.D., D.D.S., LL.B.
Boutwell, Leslie Barnes, D.M.D.
Caush, Douglas E., L.D.S.
Chaddock, Charles Gilbert, M.D.
Chittenden, Charles C., D.D.S.
Crandall, G. C., B.S., M.D.
Cryer, M. H., M.D., D.D.S.
Cumston, Charles Greene, M.D.
Curtis, G. Leroy, M.D., D.D.S.
Dawbarn, R. H. M., M.D.
Decker, Walter E., D.D.S.
Eames, George F., M.D., D.D.S.
Endelman, Julio, D.D.S.
Fine, Wm. Middleton, D.D.S.
Fitzhardinge, H. C., D.D.S.
Fletcher, M. H., M.D., D.D.S.
Franklin, Milton, M.D.
Giblin, Thomas J., D.M.D.
Gillett, Henry W., D.M.D.
Gilmer, Thomas L., M.D., D.D.S.
Grant, George F., D.M.D.
Green, Leo, A.B., D.M.D.
Hales, Leonard C., D.D.S.
Hickman, H. B., D.D.S.
Hogue, F. Wilson, D.M.D.
Janlusz, Henry J., D.D.S.
Kirk, Edward C., D.D.S., Sc.D.
Kyle, D. Braden, M.D.
Latham, V. A., M.D., D.D.S., F.R.M.S.
McCullough, P. B., D.D.S.
Marshall, John S., M.D., M.S.
Mayer, Frank R., D.D.S.
Moffatt, R. T., D.M.D.
Morgenstern, Michael.
Ottolengui, Rodrigues, M.D.S.
Peirce, C. N., D.D.S.
Pike, Charles C., D.M.D.
Potter, William L., D.M.D.
Reade, Robert J., M.D., D.D.S.
Rhein, M. L., M.D., D.D.S.
Sanger, R. M., D.D.S.
Smith, D. D., M.D., D.D.S.
Smith, Eugene H., D.M.D.

Smith, F. Milton, D.D.S.
Southwell, Charles, D.D.S.
Stanley, Ned A., D.M.D.
Storey, J. E., D.D.S.
Talbot, Eugene S., M.D., D.D.S.
Tousey, Sinclair, A.M., M.D.
Trueman, W. H., D.D.S.
Walker, William Ernest, M.D., D.D.S.
Wedelstaedt, E. H., D.D.S.
Wheeler, H. L., D.D.S.
Whitney, J. M., M.D., D.D.S.
Worman, J H.

